# Tropical surface urban heat islands in east Africa

**DOI:** 10.1038/s41598-023-31678-6

**Published:** 2023-03-18

**Authors:** Gemechu Fanta Garuma

**Affiliations:** Entoto Observatory and Research Center, Atmospheric and Climate Sciences Unit, Department of Space and Planetary Sciences at the Space Science and Geospatial Institute (SSGI), Addis Ababa, Ethiopia

**Keywords:** Climate sciences, Environmental sciences

## Abstract

The horn of Africa is susceptible to droughts because of the persistent heat waves and insufficient precipitation. The growth of urban population and built-up urban environments exacerbate the overheating problems due to the urban heat island effects. Understanding the impacts of anthropogenic activities in such dry environments is important to control or mitigate extreme heat leading to droughts. This is required to preserve soil moisture, pothole waters, lakes and rivers that are required for pasture and drinking water. Nonetheless, the intensity and duration of the urban heat island effects have not been investigated in this region resulting in the underestimation of the intensity and severity of the extreme heat events. This study therefore performs the quantitative analyses of the intensity, duration and causality of the tropical surface urban heat islands (TSUHIs) for the first time using earth observation information at a regional to local scale. It also identifies the factors that control TSUHIs, considering background climate, population, vegetation and the impervious urban fractions. Results showed that the TSUHI in the capital cities of tropical east Africa varies from 1 $^{\circ }$C in Dodoma to 4 $^{\circ }$C in Kampala and reaches up to 8 $^{\circ }$C in Khartoum. The mean temperature contribution to regional climate from 2000 to 2020 is 0.64 $^{\circ }$C during the day and 0.34 $^{\circ }$C during the night, a mean total of around 0.5 $^{\circ }$C, a 0.25 $^{\circ }$C increase per decade. This is a quarter of the increase in global surface temperature, which is $\approx$ 1.09 $^{\circ }$C from 2011 to 2020 compared to the 1850–1900 level. Most of these capital cities in this region exhibited high TSUHIs from late summer to winter and are dependent on mainly population, vegetation, evapotranspiration and soil moisture in different proportions. This urban induced additional temperature has been intensifying droughts in tropical east Africa. Therefore, urban planners are advised to consider the impacts of TSUHIs to reduce the severity of droughts in the tropical east Africa region.

## Introduction

The mean urban temperature exceeds that of rural areas in the current climate^1–3^. The urban heat island intensity is likely to increase in the future climate induced by global warming^4^. This presents a great danger to the urban communities under growing population, climate change and rapid urbanization. Many studies regarding the effects of urban heat islands have been conducted in the mid- to high latitudes^5^. Some studies have identified the urban heat islands in tropical cities, Rajagopalan et al.^6^ for Muar and Wang et al.^7^ for Kualalampur in Malaysia; Chow and Roth^8^ for Singapore; and Estoque et al.^9^ for Baguio in Philippines. There have been few isolated city scale studies in tropical sub-saharan Africa. For example, Li et al.^10^ for Kampala, Uganda; Simwanda et al.^11^ for Addis Ababa, Ethiopia, Lagos, Nigeria, Nairobi, Kenya, and Lusaka, Zambia. Nevertheless, almost all cities exhibit the urban heat island effects regardless of latitude, altitude, morphology, scale, size and background climate^12,13^.

Many studies (e.g.,^14–17^) investigated the synergistic interactions of heat waves and urban heat islands in the mid and high latitudes. In sub-saharan tropical Africa, droughts and humidity exacerbate urban warming because the synergistic interaction of urban heat islands with heat waves creates unusually hot temperatures that stay longer. However, studies linking droughts with tropical surface urban heat islands are lacking. Furthermore, global warming is increasing the average global temperature by 1.09 $^{\circ }$C in a decade from 2011 to 2020 compared to the 1850–1900 level^18,19^. The global warming trend is expected to increase the frequency and intensity of heat waves across the globe^20–22^. The impact of the global warming induced heat waves, dry spells and other extremes of temperature in the Greater Horn of Africa resulted in persistent droughts, which has been crippling the quality and availability of water, pasture and other climate related livelihoods.

Tropical urban population is projected to increase in the future^23^. Subsequently, the tropical economies are growing faster than the rest of the world implying 95% of urban expansion in the next decades will take place in the developing world^24^. These growing urban populations and the associated urban expansion are expected to create extremely warming urban environments. The elevated urban temperatures are associated with elevated rates of illnesses and deaths (e.g.,^25^). These also create stagnant weather conditions favorable to retain pollutants in few pockets of urban environments that would cause health problems (e.g.,^26^). Because of the expected population growth in urban centers, congestion of people into few places would increase the need for more water and electric power^27,28^. It also creates favorable conditions for the viral transmissions, for example, flu and covid-19^29^. Furthermore, the consequent high evaporation from urban areas can seriously affect the water availability by drying the soils and creating moisture deficits^30^. This can seriously affect the hygienic water availability that is required to reduce viral transmissions. Understanding the key drivers of urban warming in tropical cities is therefore important to reduce the urban induced weather, climate and air quality impacts.

There is a strong link between the urban surface heat islands and urban surface water, albedo , evapotranspiration and soil moisture in mid-latitude countries. Hathaway^31^ investigated the cooling effects of urban river waters in the UK; Jiang^32^ in Shanghai, China; Lin^33^ in Pearl River Delta Metropolitan Region (PRD); Gunawardena^34^ in London UK; Gunawardena^34^ in Pennsylvania, the USA document similar results. Population, background climate and the urban heat island intensity are the determining factors in urban overheating^35^. Likewise, the rate of evapotranspiration and surface reflectivity^36^ is directly proportional to the urban cooling effects based on the availability of moisture. However, such links have not been established in tropical sub-saharan cities.

This study is therefore about the quantitative analyses of the tropical urban heat islands for the first time using satellites’ earth observation information at a regional to local scale. It also investigates the association between the urban heat islands and the important determining factors, such as background climate, vegetation and the size of built-up environments. The outputs from this study are important to design extreme heat mitigation strategies for urban environments in tropical cities. It also identifies the most determining factors for such events considering population, vegetation and soil moisture in these urban environments.

The paper is organized as follows: the “Data sources and methods” Section presents the data sources and methods of data analysis; the “Results and discussion” section explains the results and discussion, and the “Conclusion” section concludes the overall outcome of the study.

## Data sources and methods

### Study locations

**Figure 1:**
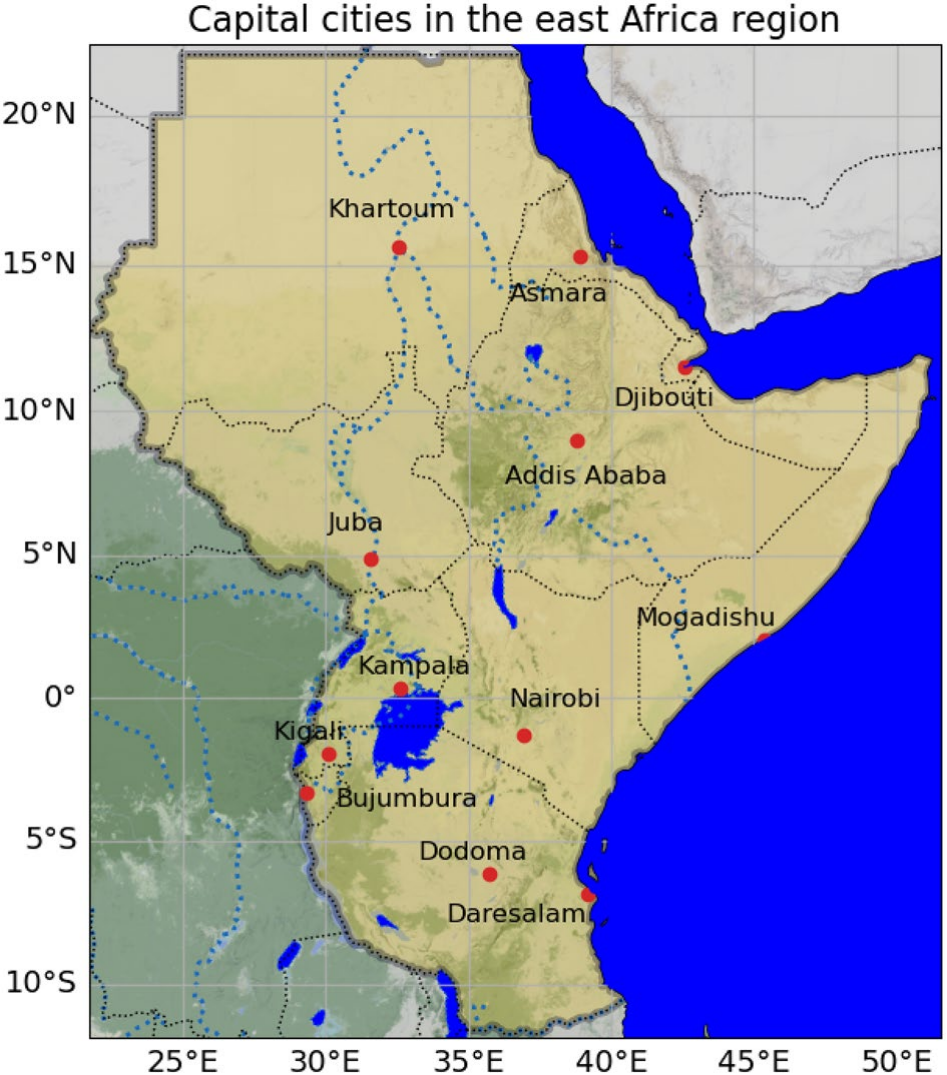
The capital cities in the east Africa region are indicated by the red circles. The region is highlighted with light gold color and all the geophysical features are blue for water and green for terrain heights.

The study focused on the capital cities in the eastern Africa region (Fig. 1). The climatology of the region varies from hot and dry desert regions in the eastern and southern parts (Somalia, Djibouti, eastern and south eastern Ethiopia, Sudan and northern Kenya) to cooler and wetter areas (central and southern Kenya, South Sudan, Uganda, Rwanda, Burundi, Bujumbura and Tanzania)^37^. The region has complex terrains from lowlands in the eastern part of the region to mountainous regions in the western highlands of Ethiopia. In this study, the urban heat island characteristics of the capital cities in the region are determined. The complex topographical characteristics and versatile climatology in the region makes the isolated contribution of the urban heat islands to the warming of the region challenging.

### Data sources

#### Urban land fraction and land surface temperature

**Figure 2:**
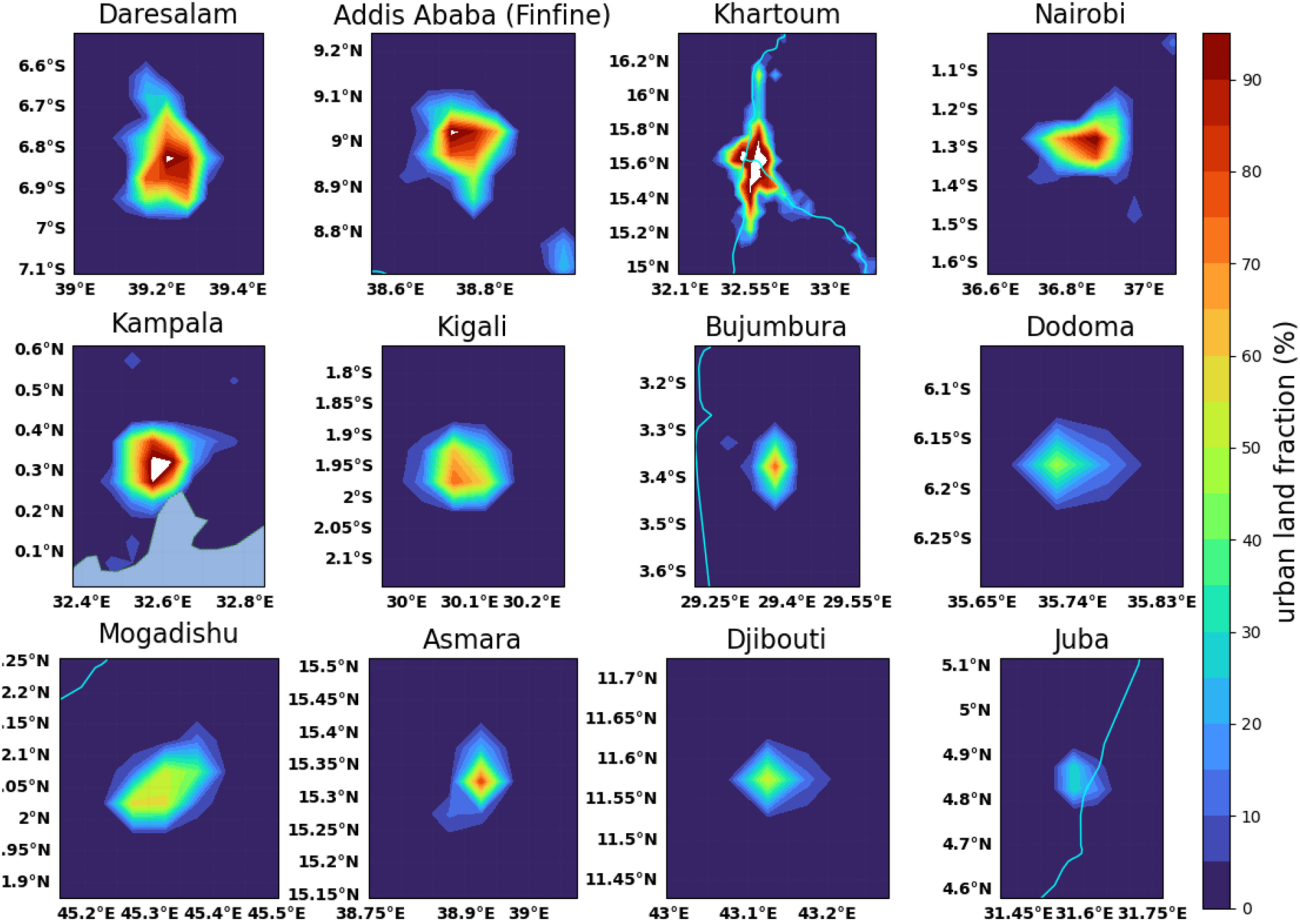
Urban land fraction around the capital cities in the greater horn of Africa region.

The urban land fractions for each of the cities in the GHA are dependent on the urban size and population density (Fig. 2). In this study, the land use fractions are used to filter out data for the urban areas. That is identifying urban and the surrounding rural areas is performed by using the urban land fractions. As such, the grid with urban land fraction of greater than 0.5 (50% of the land fraction) is taken as urban area and the grid with less than or equal to 0.5 (< 50% of the total land fraction) is rural. This is considering the idea that if the majority of the land fraction is urban, there is a high probability that the area in which this grid lies is urban. The neighboring grids were also taken into account. That is if the neighboring grids have more urban fractions, then the grids are taken as grids falling in urban areas. The data was obtained from the Moderate Resolution Imaging Spectroradiometer (MODIS). Both the MODIS land surface temperature^38^ and urban land fractions^39^ are available with a horizontal resolution of 0.05° daily and monthly. The data from 2000 to 2020 was used in this study.

The land surface temperature data is used to compute the tropical surface urban heat islands. Bias correction of the MODIS land surface temperature comparing it with the surface level station data from each of the capital cities in the Greater Horn of Africa Region was performed.

#### Surface albedo and water distribution

Surface albedo is important to quantify the fraction of radiation reflected from the surface of the Earth. Reflective capacity of urban land surfaces is important to understand the surface absorption and reflection factors. The surface albedo data is obtained from SPOT/VGT 1km Collection 2 and PROBA-V 1 km Collection 1 data at 0.5° × 0.5° horizontal resolution. This surface albedo data was interpolated to the land cover fraction resolution before it is used in this study.

The land-water distribution was obtained from the Space Shuttle Topographic Mission (SRTM) and MODIS^40^. This land water distribution is available from 2000 to 2015 yearly with a spatial resolution of 250 m $\times$ 250 m. This data quality was checked through comparison with in situ observation and independent finer solved air-and space borne sensors. This data was interpolated to the land surface fraction resolution data before it was used.

#### Observation data

The temperature data from the meteorological stations was obtained from the Berkeley Earth Data https://berkeleyearth.org/data/. This data was used to correct the bias and validate the data from the MODIS Earth Observation data. This study also used population data to identify the dominant factor in the tropical surface urban heat islands. The population data was obtained from World Population Review, https://worldpopulationreview.com/.

#### Soil moisture and evapotranspiration

Soil moisture is an important factor that influences the partitioning of the available energy at the urban and rural surfaces. If the moisture content of the soil is high, there is a high evapotranspiration rate. As such, soil moisture determines the amount of energy exchanges between the land and the atmosphere. Evaporation and transpiration as cooling background factors were also used. For soil moisture, evaporation, transpiration and all the related evaporation fields, the Global Land Evaporation Amsterdam Model (GLEM)^41–43^ data was utilized. The data is available daily and monthly from 1980 to 2021 with a spatial resolution of 0.25$^{\circ }$ $\times$ 25$^{\circ }$ climate modeling grids. This data was interpolated to the land cover fraction resolution before it was used in this study.

### Methodology

#### Quantification of tropical surface urban heat islands

There are several methods to investigate and understand the impacts of urban environments on weather, climate and air quality. The most frequently used method is comparing urban and non-urban (rural) mean values of the meteorological variables (e.g.,^4^). This is based on the current urban development scenario and considering how this built environment affects weather/climate compared to the current rural environment. It can be achieved in three possible ways, (i) comparing the mean variables of the grids in the dense urban areas and grids in the rural areas outside of the city. The two considered grids for comparison should differ in the type of land cover, that is natural land surfaces (e.g. forests, bareground, grass, water, etc) for rural and built-up for urban environments (e.g., buildings and roads), (ii) comparing the mean temperatures of the selected urban grids in the city center and of the selected rural grids in all the directions of the surrounding areas, and (iii) comparing the same grid cell, i.e., one with built-up surfaces and the other by omitting the urban surfaces.

The less frequently used but very important method is comparing past, present and future evolution of urbanization (e.g.,^44^), implementing the urban evolution into climate models and comparing how the meteorological variables evolve in time with the urban developments. This can be performed in particular considering the land use transition from urban to rural and in the rare occasions, from rural to urban (e.g., in the case of urban fires). This requires different input fields (e.g., building characteristics, building heights, widths, composition, etc) for each land use transition. Land use transition by itself is a scientific challenge because it requires historical information which rarely exists. So, combining all the required input fields for each transition and performing analyses to isolate the impacts of the human built environment on weather, climate and air quality is complex but important.

The other rarely used approach is comparing the climatic condition before the city is built with that after the city is built. This can be done by employing the opportunity when new cities are designed and built. The scenario during fire damage and re-building can be used as an opportunity for this particular approach. At a local/neighborhood scale, real estate building sites, condos and other commercial and recreational areas before and after the construction can also be used to quantify the urban impacts on weather or climate.

The other method is based on scenarios of building fractions, road fractions, impervious fractions, vegetation fractions, solar panel fractions (future roofs are expected to be covered partially by solar panels). This is required to compare past, present and future impacts of urbanization. The scenarios could be divided into a few categories, e.g., low urban development, medium, and high urban development areas or the Local Climate Zones^12^. Hypothetical fractional changes depending on the above scenarios could provide insights into the interactions of these different Land Use and Land Cover (LULC) changes. Further approach is based on anthropogenic heat and pollutants emissions. Heat, moisture fluxes and pollutant emissions scenarios can be used to investigate the impacts of urbanization into past, present and future climate.

Comparing weekdays and weekends can also be used. This method is used specifically to investigate the impacts of anthropogenic heating (AH) because during the weekdays people use vehicles to commute to work and the associated anthropogenic heat and pollutants emissions from traffic have an impact on the local/regional weather and climate. Similarly, comparing mornings before solar radiation affects the local meteorological variables with the variables later in the day. It is assumed that the thermodynamic influence of built urban environments during early morning is almost zero. Because of the differences in light emission, absorption, scattering, shadowing and radiation trapping effects that come later during the day when the solar radiation is higher above the horizon, the urban influence can be quantitatively obtained.

The most known urban heat islands (UHIs) are the mean temperature difference between urban and rural air temperatures. The urban heat islands computed using the land surface temperatures (LSTs) difference between urban and rural areas is known as the surface urban heat islands (SUHI). The other type of urban heat islands are computed using the temperature difference between the ground level and the mean height of roof tops and urban tree tops. The types of urban heat islands are discussed in Stewart et al.^45^. This study uses the land surface temperatures to compute the urban heat islands, hence named as the tropical surface urban heat islands (TSUHI) to differentiate it from heat islands in the higher latitudes.

The qualitative spatial analysis and quantitative mean value temporal analyses are used in this study to identify the intensity of the TSUHIs. The spatial distribution of the land surface temperature with shaded contours and lines are used to understand the spatial TSUHIs. The closed contour loops identify the TSUHIs or the tropical surface urban cool islands (TSUCI) depending up on whether the land surface temperature is higher/lower in the cities compared to the rural areas. Furthermore, area averaged seasonal cycle of the TSUHI/TSUCI is used for the temporal analysis. To identify the urban and rural grids the fraction of vegetation and built-up urban surfaces are used (Fig. 2). That is if the grids have more urban fractions (> 50%) and less natural fractions (< 50%), it is identified as urban and the surrounding area with less urban fractions (< 50%) and more vegetation fraction (> 50%) are used as rural area. This method identifies almost all grids in the center of the cities as urban and the surrounding grids as rural (Fig. 2). Therefore, the TSUHI is given by

1 $$\begin{aligned} TSUHI_{d/n} = LST_{d/n,U} - LST_{d/n,R} \end{aligned}$$

where the $TSUHI_{d/n}$ is the tropical surface urban heat island for day/night, $LST_{d/n,U}$ is the land surface temperature for day/night in the urban area, and $LST_{d/n,R}$ is the land surface temperature for day/night in the rural neighborhood. If TSUHI is negative, it is the tropical surface urban cool island (TSUCI).

#### Bias correction

Like any satellite data, the MODIS land surface temperature shows systematic bias when compared to station data. Therefore, the bias from this data was corrected against observation from the nearest meteorological station. That is, the LST values from the model were calculated to the nearest grid point to the stations before statistical analysis was performed. However, the station’s data was found to contain few outliers and these were filtered out by using the median filter method^46^. Once the outlier filtering was completed this data was used to correct the bias from the MODIS data. After a few curve fitting attempts, the best fit to remove the bias suitable for all the stations were achieved. The bias correction equation obtained is taking the mean value differences between the MODIS and observation data and adding it to the MODIS data, that is

2 $$\begin{aligned} T_{BC}(t) = T_{M}(t) + (\overline{T_{O}} -\overline{T_{M}}) \end{aligned}$$

where the $T_{BC}$, $T_{M}$ and $T_{O}$ are the bias corrected (BC) land surface temperature (LST), the MODIS LST and station observation temperatures respectively. To obtain pattern similarity between the observation LST ($T_{O}$) and the bias-corrected MODIS LST ($T_{M}$), the statistical correlation coefficient was computed. The correlation coefficient between the LST variable for MODIS and observation is

3 $$\begin{aligned} R = \frac{\frac{1}{N}\sum _{n=1}^{N} (T_{M} - \overline{T_{M}}) (T_{O} -\overline{T_{O}})}{\sigma _{T_{M}}\sigma _{T_{O}}}, \end{aligned}$$

where $\overline{T_{M}}$ and $\overline{T_{O}}$ are the mean values and $\sigma _{T_{M}}$ and $\sigma _{T_{O}}$ are their respective standard deviations. The correlation coefficient reaches a maximum value of 1 when the two datasets have the same centered pattern, otherwise the R values are less than 1.

## Results and discussion

### Bias correction of MODIS land surface temperature

**Figure 3:**
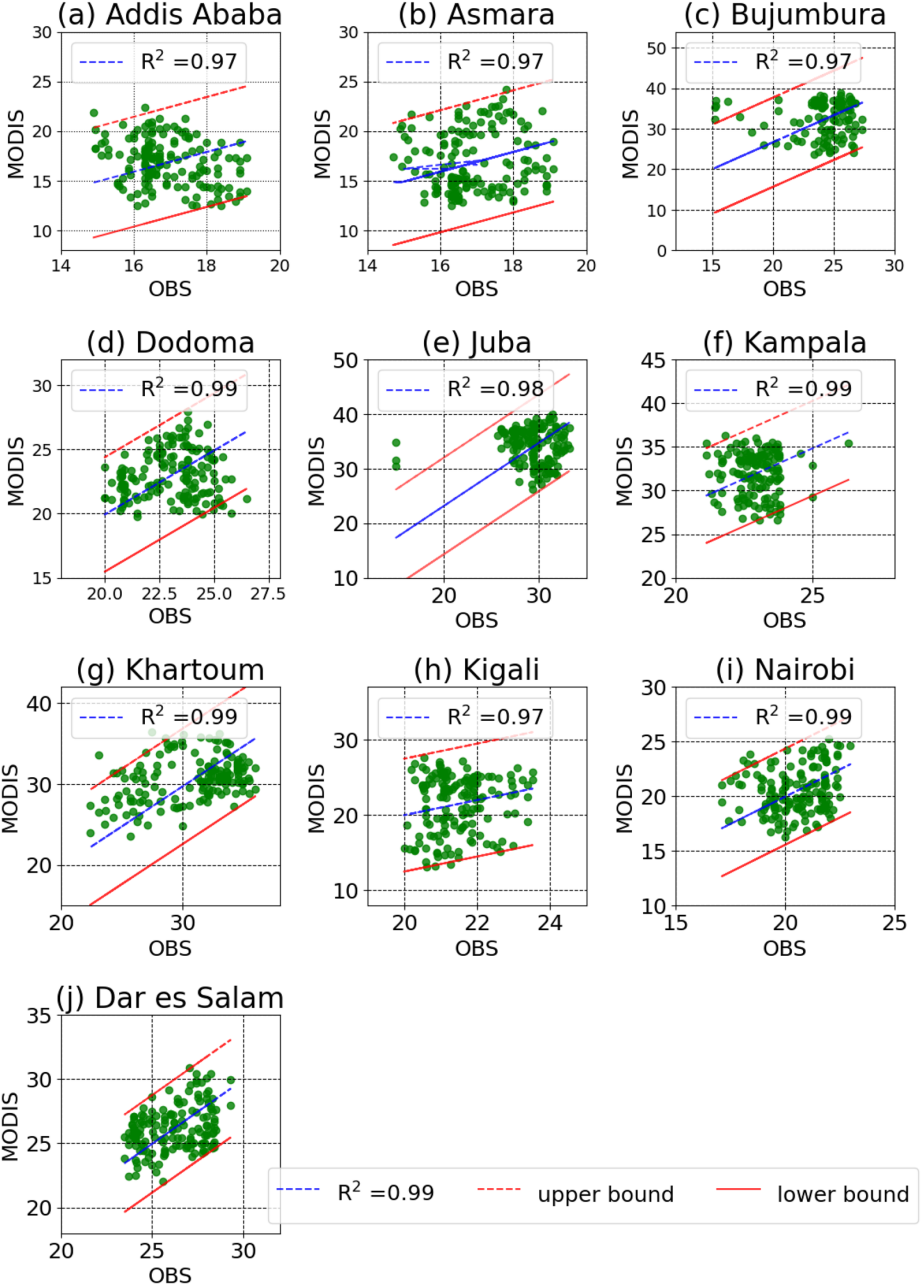
Validation of the land surface temperature from the Moderate Resolution Spectroradiometer (MODIS). The upper and lower bounds are shown with respect to the 95% interval. These are the stations where observation data is available. Data is not available for the two other stations (Djibouti and Mogadishu) considered in the study.

Information from the stations were used to improve the MODIS data using systematic bias correction technique (Eq. 1). The bias corrected data was found to correlate strongly to the stations data with R-squared values greater than 0.97 for all the stations in the capital cities in the east African region (Fig. 3). The performance of the MODIS data for the cities Addis Ababa, Asmara, Bujumbura and Kigali is in the upper lower level with an R-squared value of 0.97 each. The capital of South Sudan, Juba has an upper intermediate performance with an R-squared value of 0.98 and the other capital cities have an absolute upper level performance with an R-squared value of 0.99. The upper and lower bounds with a 95%/5% confidence level showed that there are only few data points out of these bounds. This implies that the Bias corrected LST has shown satisfactory performance that qualifies it for the assessment of urban heat islands in these tropical capital cities.

### Tropical surface urban heat islands (TSUHIs)

#### Spatial TSUHIs

**Figure 4:**
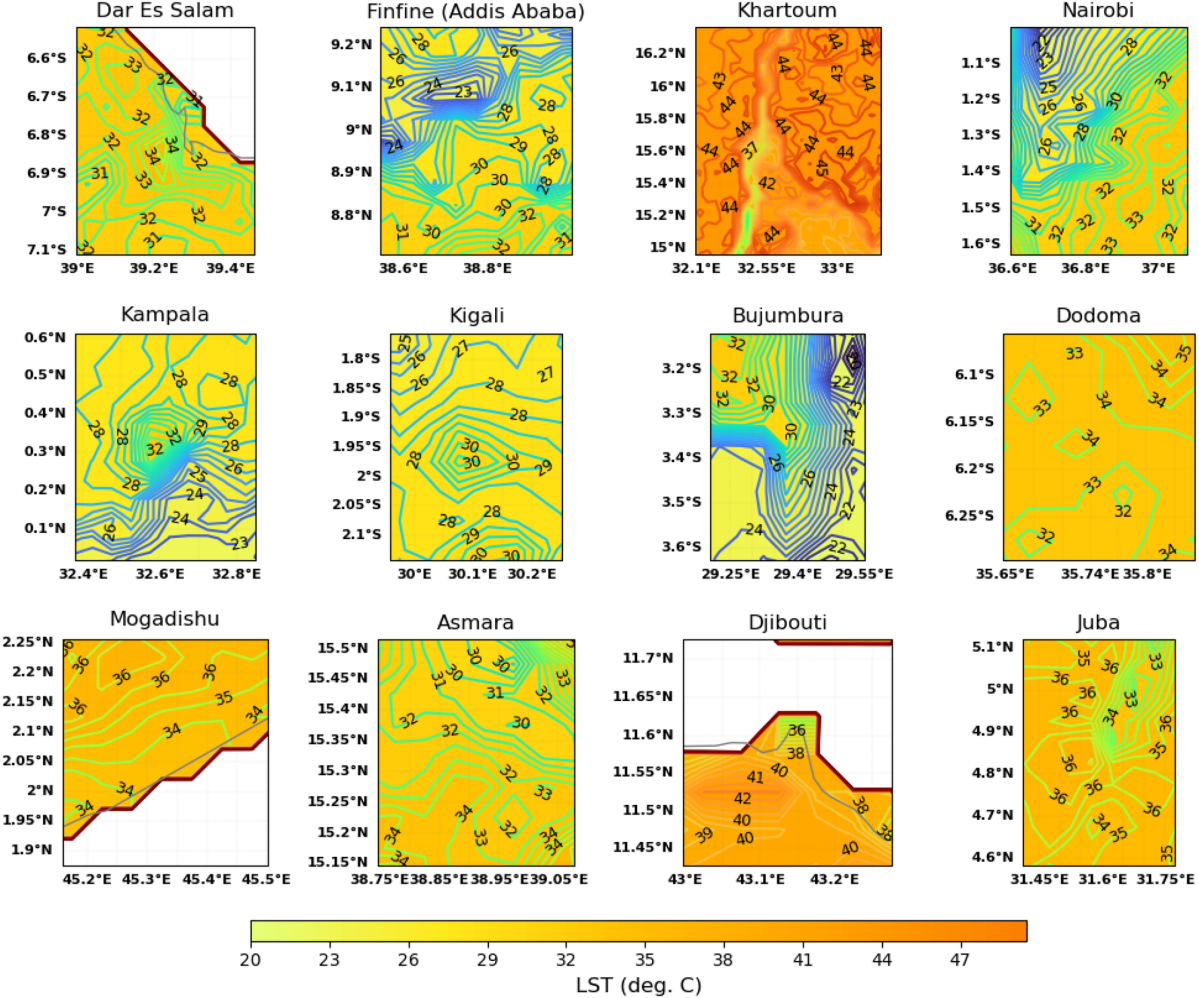
Land surface temperature around the capital cities in the greater horn of Africa region.

**Figure 5:**
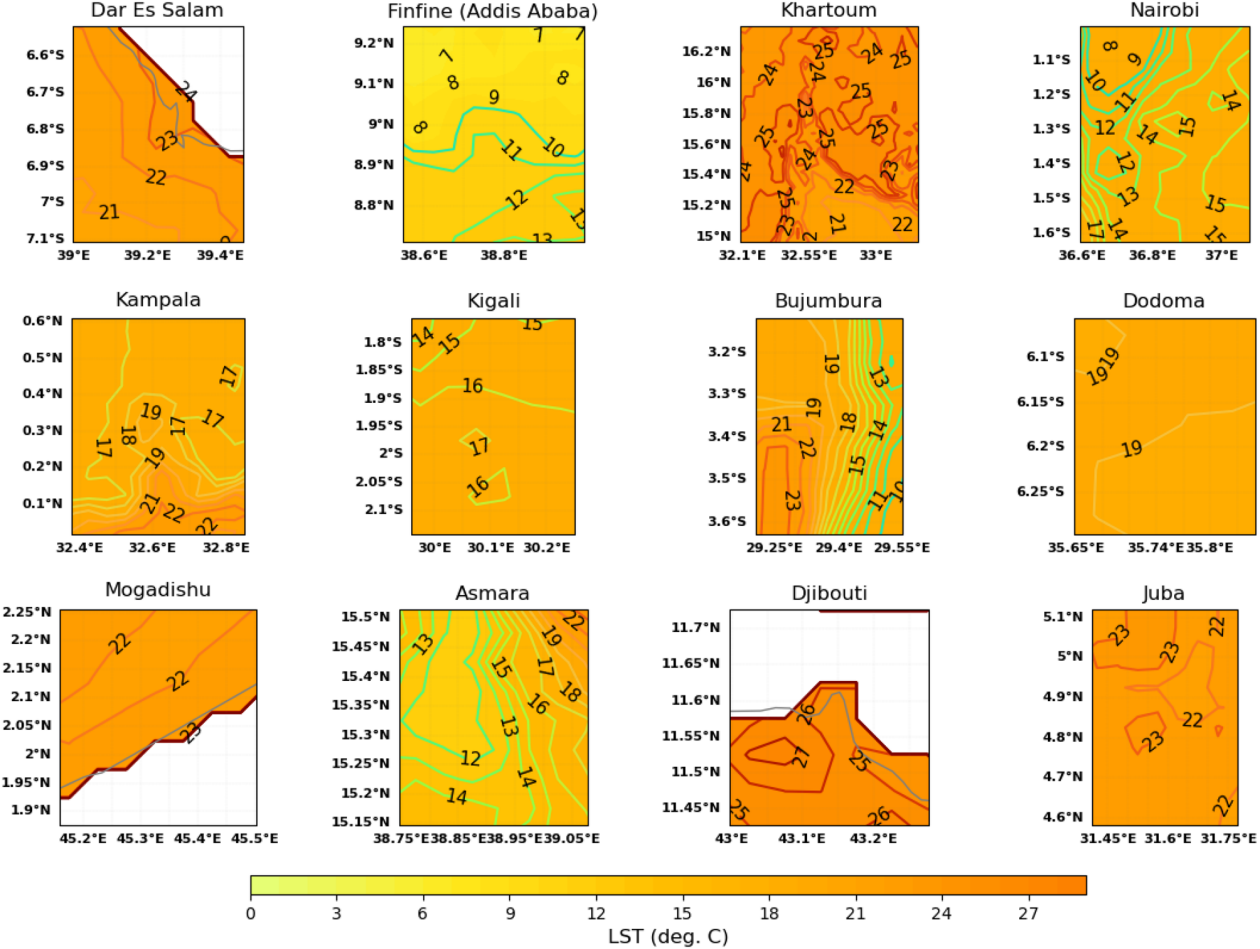
Night time land surface temperature around the capital cities in the greater horn Africa.

Results showed most of the cities in tropical east Africa exhibited surface urban heat islands both during the day and night (Figs. 4 and 5). This is considering the temperature contour lines in the center of the cities (compare Figs. 2, 4 and 5) with the contour lines surrounding the center of the cities. The urban heat islands for each of the cities have different spatial features. The first category of the cities have distinct urban heat islands at the center of the cities shown by closed contour lines. The cities with this characteristics are Daresalam, Kampala, Kigali, and Dodoma during the day. Daresalam has a heat island amounting to 2 $^{\circ }$C (34 $^{\circ }$C–32 $^{\circ }$C); Kampala has 4 $^{\circ }$C (32 $^{\circ }$C–28 $^{\circ }$C); Kigali has 2 $^{\circ }$C (30 $^{\circ }$C–28 $^{\circ }$C), and Dodoma has 1 $^{\circ }$C (34 $^{\circ }$C–33 $^{\circ }$C).

Cities that have water bodies have heat islands to the opposite side, for example, Khartoum, Mogadishu and Djibouti. The Nile river in Khartoum seems to cool settlements around the river to the eastern and western sides of the city. The lowest temperature near the river is 37 $^{\circ }$C followed by 42 $^{\circ }$C and 44 $^{\circ }$C–45 $^{\circ }$C further to the west and east respectively (Fig. 4). The heat island for Khartoum, therefore, ranges from 7 to 8 $^{\circ }$C, that is the difference is 7 $^{\circ }$C in some places where the temperature is 44 $^{\circ }$C and 8 $^{\circ }$C in places where the temperature is 45 $^{\circ }$C. This implies that the heat island for Khartoum is highest because the temperature in the city is higher further away from the river and lower near the river because of the cooling effect of the river-breeze from the Nile River. Mogadishu is near to the Indian Ocean and the temperature difference near the ocean water and further up the latitude near the center of the city is 2 $^{\circ }$C; for Djibouti the heat island is around 6 $^{\circ }$C (42 $^{\circ }$C at the center of the city to 36 $^{\circ }$C near the red sea).

The third category of the cities: Addis Ababa, Nairobi, Bujumbura, Asmara and Juba have non-uniform temperature distribution in and around the cities. The cities in this category are highly susceptible to inhomogeneous distribution of urban heat islands as a result of the non-uniform distribution of the buildings and roads, complex landscapes and density of urban population. It is quite difficult to accurately identify the intensity of the surface urban heat islands in these cities.

The nighttime tropical surface urban heat island intensity is lower than the daytime TSUHIs (compare Figs. 4 and 5). Only few cities showed distinct closed contours of the TSUHIs. These cities are Nairobi, Kampala, Kigali, Djibouti and Juba (Fig. 5). This nighttime TSUHI reaches a maximum of 2 $^{\circ }$C, for example, Nairobi, Kampala and Djibouti. The nighttime TSUHI for Kigali and Juba is 1 $^{\circ }$C. Asmara showed a negative TSUHI or a positive TSUCI amounting to $-6\,^{\circ }$C to the western side of the city. This is expected because of the sea-breeze from the red sea on the right side, which advects warmer air to the Asmara city during the night. However, it is not obvious from the spatial land surface distribution information alone to determine the nighttime TSUHIs for the cities: Daresalam, Addis Ababa (Finfine), Khartoum, Bujumbura, Dodoma and Mogadishu. It is also difficult to understand the urban heat island characteristics during the day for some cities with inhomegeneous distribution of TSUHIs because of the complex urban development in the tropical cities. That is few high rise buildings are located around houses that are thatched roofs or slums. Therefore, it is necessary to calculate the mean total urban area temperature and compare it with the surface temperature in the rural areas surrounding the capital cities, to understand the intensity of the TSUHIs. This is explained in the next section.

#### Temporal TSUHIs

**Figure 6:**
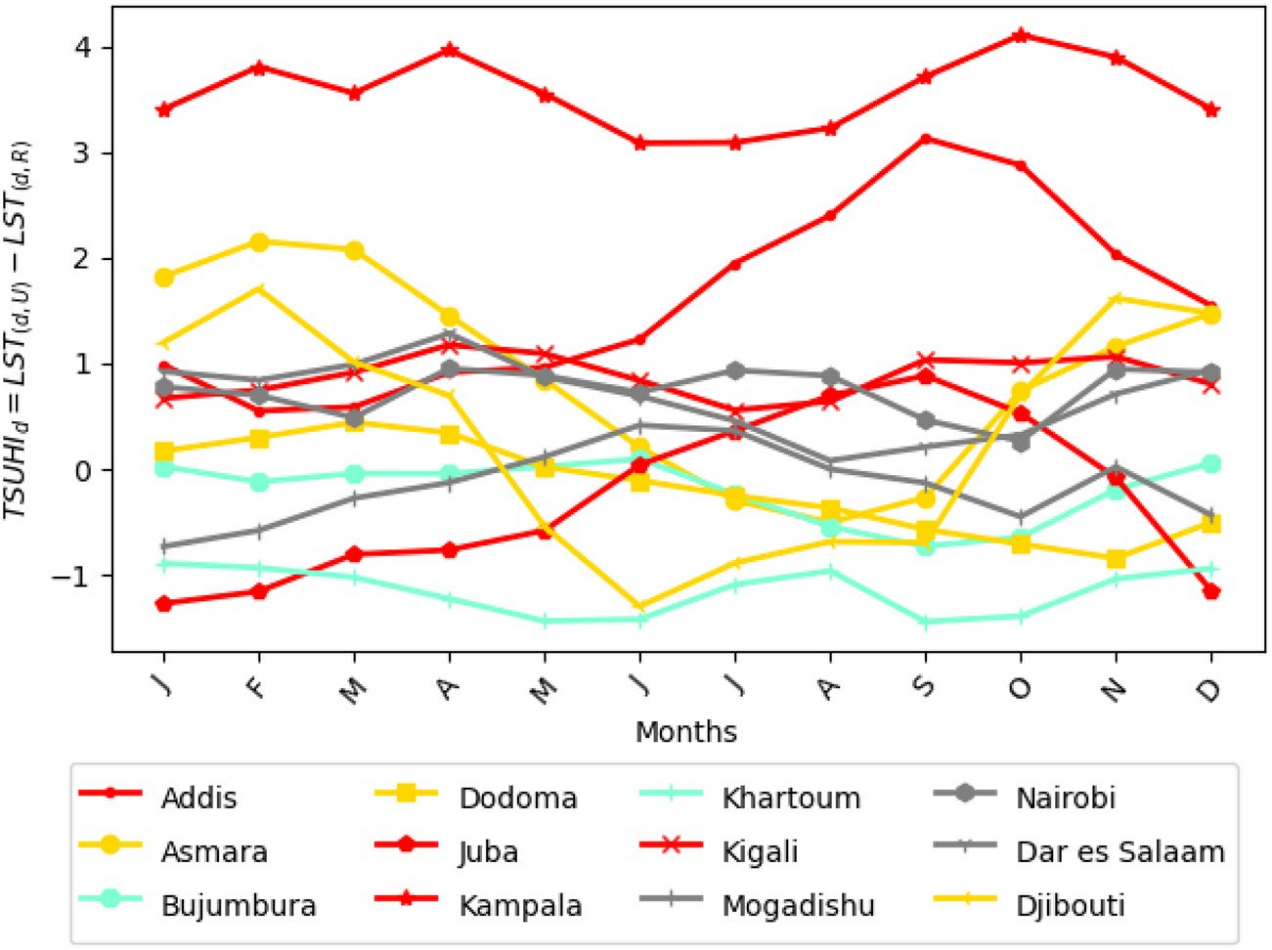
Seasonal cycle of tropical surface urban heat islands (TSUHIs) in the greater horn of Africa during the day. Different markers are used to differentiate the lines and the same colors are used for cities identified to have the same seasonal characteristics of the tropical surface urban heat islands.

The area averaged monthly mean of the urban heat islands show the seasonal intensity of the tropical surface urban heat islands (Fig. 6). The seasonal urban heat island characteristics is inhomogeneous across the cities in the greater horn of Africa region. Most of the cities have shown to have high intensity TSUHI from late summer to winter. The cities with high TSUHI in late summer and early autumn, that is August-September-October-November (ASON) are Kampala, Addis Ababa, Juba and Kigali (red lines in Fig. 6); cities with late autumn and winter (Novermber-December-January-February, NDJF) are Djibouti, Dodoma and Asmara (yellow lines in Fig. 6); cities with non-seasonal urban heat islands are Mogadishu, Nairobi, and Daresalam (gray lines in Fig. 6). Bujumbura and Khartoum do not have positive area averaged monthly mean urban heat islands (lines with aquamarine color in Fig. 6). Of the 12 cities considered in this study 10 have shown to have positive mean area tropical surface urban heat islands. This implies that most of the cities in the greater horn of Africa do exhibit the tropical surface urban heat islands (TSUHIs). Kampala has the highest urban area average TSUHI followed by Addis Ababa which showed highly seasonal TSUHI. Khartoum is the only city with the area averaged tropical surface urban cool islands (TSUCI). Even though, Khartoum has a highest ($\approx\,8\,^{\circ }$C) tropical surface urban heat islands (TSUHIs) considering the spatial analyses as shown in “Spatial TSUHIs” Section , where the land surface temperature is 37 $^{\circ }$C near the Nile river and 45 $^{\circ }$C further away from the river horizontally (Fig. 4), the area average monthly mean TSUHI is negative, swinging around $-1\,^{\circ }$C (Fig. 6). This is because the maximum temperature values away from the Nile river cancel out the minimum temperature values around the river, hence the mean area TSUHI for Khartoum is almost negligible. This shows that, for some urban areas, the mean area values of the urban heat islands do not give accurate representation of the intensity of the urban heat islands. Nonetheless, it provides an estimation of how much the urban area contributes to a regional warming.

**Figure 7:**
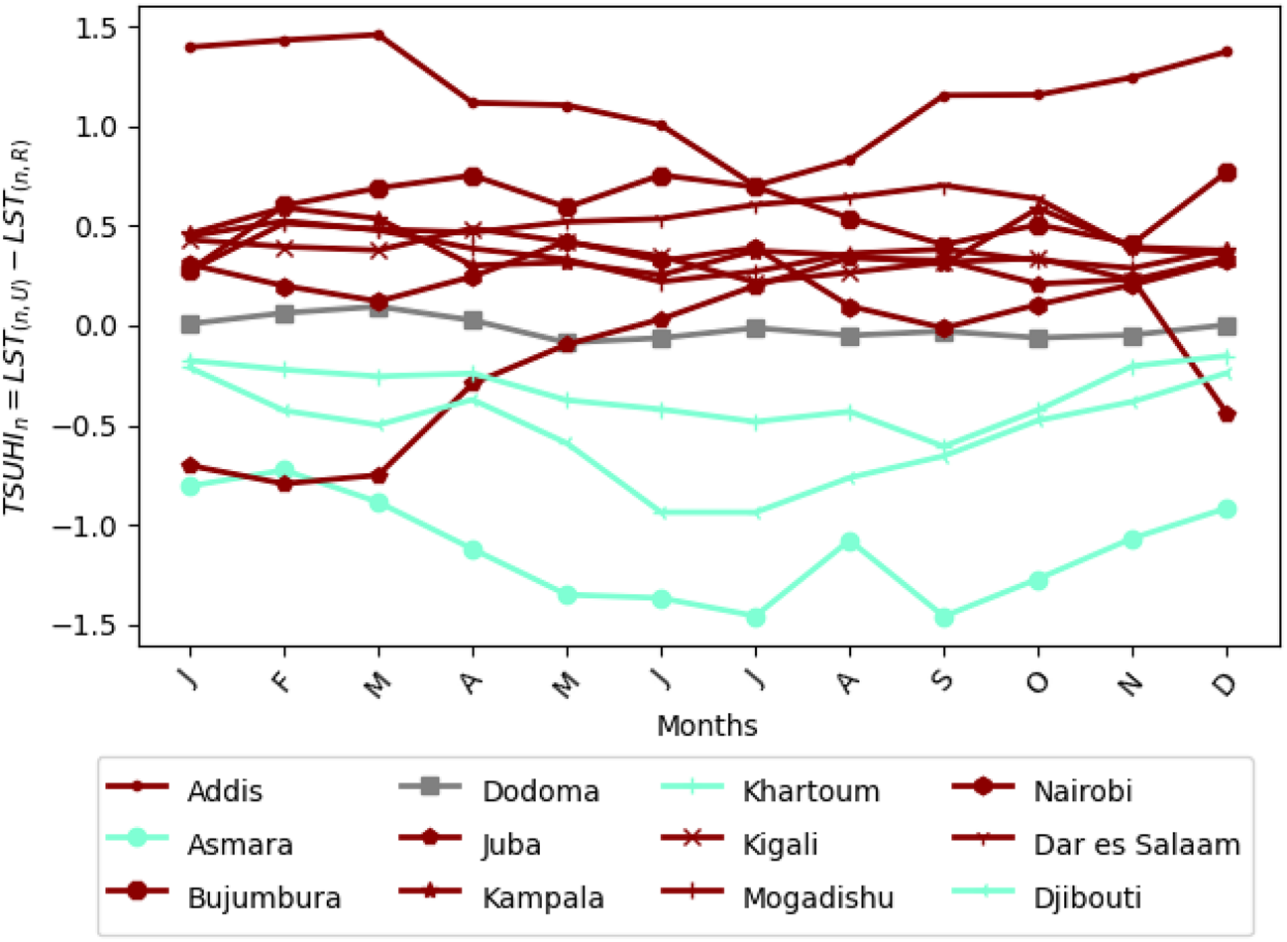
Seasonal cycle of tropical surface urban heat islands (TSUHIs) in the greater horn of Africa during the night. Cities with the night time heat islands are shown with red lines, with no observed night time heat islands are shown by the gray line, and with the night time cool islands are shown with the aquamarine lines.

The night time TSUHI is lower than the day time TSUHI (compare Figs. 6 and 7). During the night, eight of the 12 cities (Addis Ababa, Bujumbura, Daresalam, Kigali, Mogadishu, Juba, Kampala, Nairobi) have TSUHI (dark red lines in Fig. 7). Dodoma doesn’t have night time TSUHI (gray lines in Fig. 7). The rest of the cities (Khartoum, Djibouti and Asmara) have night time tropical surface urban cool islands (TSUCI) (aquamarine lines in Fig. 7). That is the cities with TSUCI have lower night time urban temperatures compared to the rural areas. This is as a result that these cities are situated near the ocean waters where cooler air flows to the cities.

The seasonal night and day time TSUHIs are not sufficient to document the TSUHI contributions to the tropical urban climate. For this reason, the total time mean of the area averaged TSUHIs was computed.

**Figure 8:**
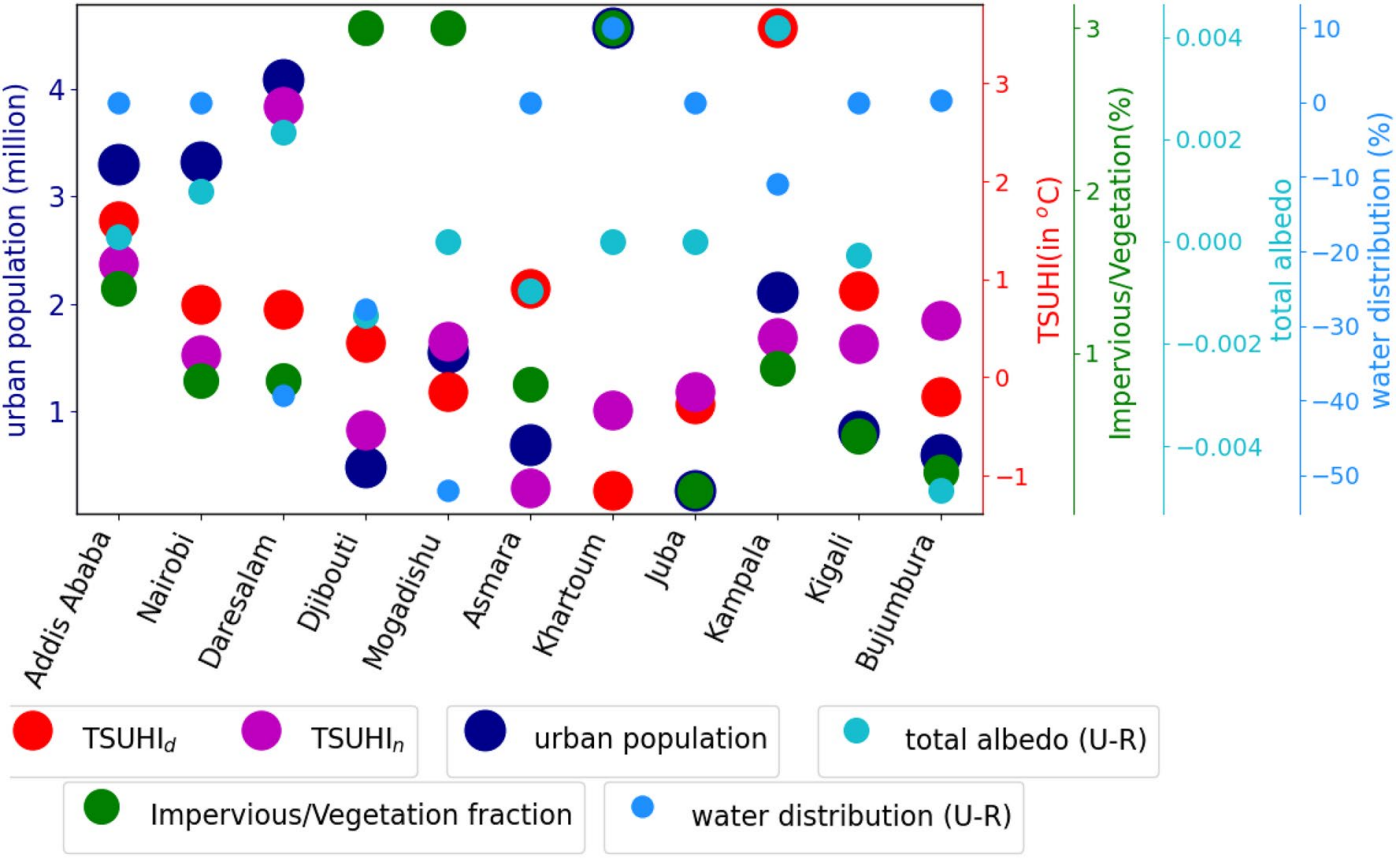
Urban factors affecting the surface urban heat islands in the Greater Horn of Africa (GHA) region. Different sizes and colors of circular objects are used to represent the different variables. The large circles with the red and pink colors represent tropical surface urban heat islands during the day and night respectively, the circles with a blue color represents the total urban population, the circles with aquamarine color represents the total albedo difference between urban and rural areas, the circles with the green colors represent the ratio of imperviousness to vegetation fractions in the urban area, and the light blue colors represent the surface water distribution difference between the urban and rural areas. The y-axis line colors and labels have similar colors with the circles representing each of the variables. The y-axis for TSUHI$_{n}$, impervious/vegetation fractions, total albedo and water distribution are shown on the right side. The x-axis shows the corresponding capital cities in the east Africa region.

The all time mean TSUHIs indicate that the contribution of the tropical urban heat islands to the mean climate of the tropical region. Accordingly, 10 of the 12 cities (Addis Ababa, Nairobi, Daresalam, Djibouti, Mogadishu, Asmara, Kampala, Kigali and Bujumbura) have positive contribution to the regional urban climate (Fig. 8) during the day. About 8 of these cities have shown to contribute positively to the regional climate during the night. For two cities, Khartoum and Juba, the mean total contribution of urban heat islands is less than zero. The maximum mean urban heat island is 4.8 $^{\circ }$C for Kampala during the day and 3.9 $^{\circ }$C for Daresalam during the night.

Regionally, the mean temperature contribution to the regional climate is 0.64 $^{\circ }$C during the day and 0.34 $^{\circ }$C during the night implying a total regional mean of 0.49 $^{\circ }$C. This implies that in the topical Africa, the urban heat islands worsen the dry urban and rural environments and exacerbate droughts. This additional regional temperature evaporates moisture from the soil and land surfaces intensifying the regional droughts. This result is a contradiction to the widely accepted assumption that tropical cities have lower contribution to the warming of the local environments. This assumption came as a result of the composition of the buildings (e.g., mud walls and corrugated iron roofs), and the high level of slams in the cities. Nevertheless, the cause of the tropical Africa surface urban heat islands is not well known. To understand the causes of these tropical urban heat islands, the relationship between TSUHIs and the surface factors such as impervious (buildings and roads) surfaces, vegetation fraction, urban population, total surface reflectivity, and the background climate factors such as evapotranspiration, soil moisture, and potential evaporation are investigated in the next section.

### Factors affecting TSUHIs

The main factors that are assumed to control the tropical surface urban heat islands (TSUHIs) can be categorized into the surface factors: population, fraction of impervious surfaces, vegetation, surface water distribution and background climate: soil moisture, evapotranspiration, total surface albedo and tropical vegetation. These factors have positive or negative temperature feedback. For example, it is assumed that high number of urban population and impervious surfaces (e.g., buildings and roads) increase the local temperature, while the presence of vegetation, surface water, evapotransipiration, soil moisture, total surface albedo, and vegetation are assumed to lower the TSUHIs. For this particular study, these determining factors are divided into two categories: the surface factors and background climate presented in the next subsections.

#### Surface factors

The surface factors that have a direct impact on tropical surface urban heat islands are urban population density, impervious and vegetation fractions, total albedo, and surface water distribution. Urban population and impervious fractions have a direct and positive relationship with tropical surface urban heat islands (e.g.,^47^). Total surface albedo and surface water have an inverse relationship with the tropical surface urban heat islands (e.g.,^48,49^).

Urban population has a direct link to urban climate warming in most of the cities. Daytime TSUHI is highly related to population in Addis Ababa, Nairobi, Asmara, Khartoum and Kampala (Figs. 8 and 11a). Figure 8 shows that an area averaged and time mean of TSUHIs in red and pink respectively for day and night compared to urban population, ratio of impervious and vegetation, total albedo and water distribution. This qualitative analysis is complemented by the quantitative correlation analysis (Fig. 11a). The correlation between TSUHIs and the determining factors such as population, imperviousness fractions, vegetation fractions, surface albedo, evaporation, transpiration, potential evaporation, soil moisture, interception loss and evaporation stresses were performed. Both the qualitative and R-squared values show that there is a strong association between population and TSUHIs. That is low/high urban heat islands are related to low/high urban population respectively. High correlation observed for cities with high urban populations, for example in Addis Ababa, Nairobi, Asmara, Khartoum and Kampala (Fig. 11a). On the other hand, low correlation is observed for the cities, Djibouti, Mogadishu, Juba and Bujumbura, where the urban population are low (Figs. 8 and 11). In these cities, the ratio of impervious surfaces to vegetation, total albedo and water distribution have a high impact on the surface urban heat islands except for Mogadishu where a very small correlation is observed (Fig. 11a).

Impervious surfaces amplify the TSUHIs, but vegetation reduces it. The ratio of imperviousness to vegetation fractions shows the level of contribution from the two combined factors. Even though, Djibouti and Mogadishu have high impervious fractions compared to vegetation, the contribution from this impervious urban fraction to TSUHI is low (Fig. 8). On the other hand, Kampala’s impervious fraction compared to vegetation is relatively low, but the day time surface urban heat island (UHI$_{d}$) in this location is relatively high. This implies that vegetation fraction plays a major role in this city compared to the other factors.

Reflection of surface radiation has a potential cooling effect in urban areas. As such, Mogadishu, Khartoum, and Juba are relatively cooler because of the surface albedo than the other cities (Fig. 8). High correlation is also observed between the TSUHIs and surface albedo for most of the cities (Fig. 11a and b). Few cities have shown weak relationship between TSUHI and albedo. These are Djibouti, Juba and Bujumbura during the day (Fig. 11a) and Juba only during the night (Fig. 11b). This is attributed to the soil properties of this tropical cities.

Water distribution has a cooling effect on the urban heat islands. This implies that cities with water availability nearby: Asmara, Juba and Bujumbura are relatively cooler (Fig. 8). However, due to the constant nature of the surface water distribution, the mean values didn’t show strong correlation with TSUHIs (Fig. 11a and b).

#### The background climate

**Figure 9:**
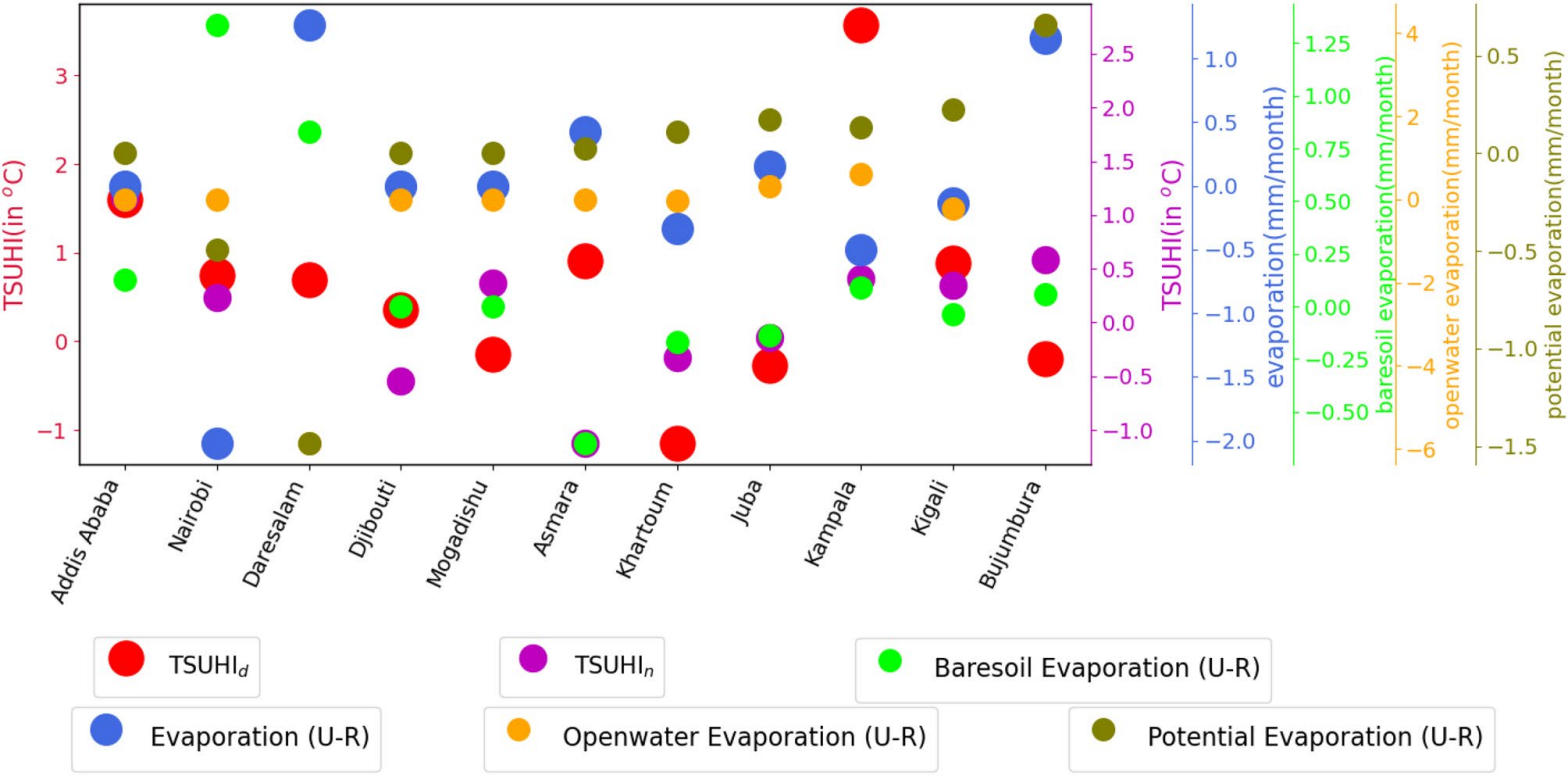
Urban evaporation affecting the tropical surface urban heat islands (TSUHIs) in the Greater Horn of Africa (GHA) region. Different sizes and colors of circular objects are used to represent the different variables. The large circles with the red and pink colors represent tropical surface urban heat islands during the day and night respectively, the blue, orange, light green and dark green colors represent the total, open water, bare soil, and potential evaporation differences between urban and rural areas respectively. The y-axis line colors and labels have similar colors with the circles representing each of the variables. The y-axis for TSUHI$_{n}$, total, bare soil, open water and potential evaporation are shown on the right side. The x-axis shows the corresponding capital cities in the east Africa region.

**Figure 10:**
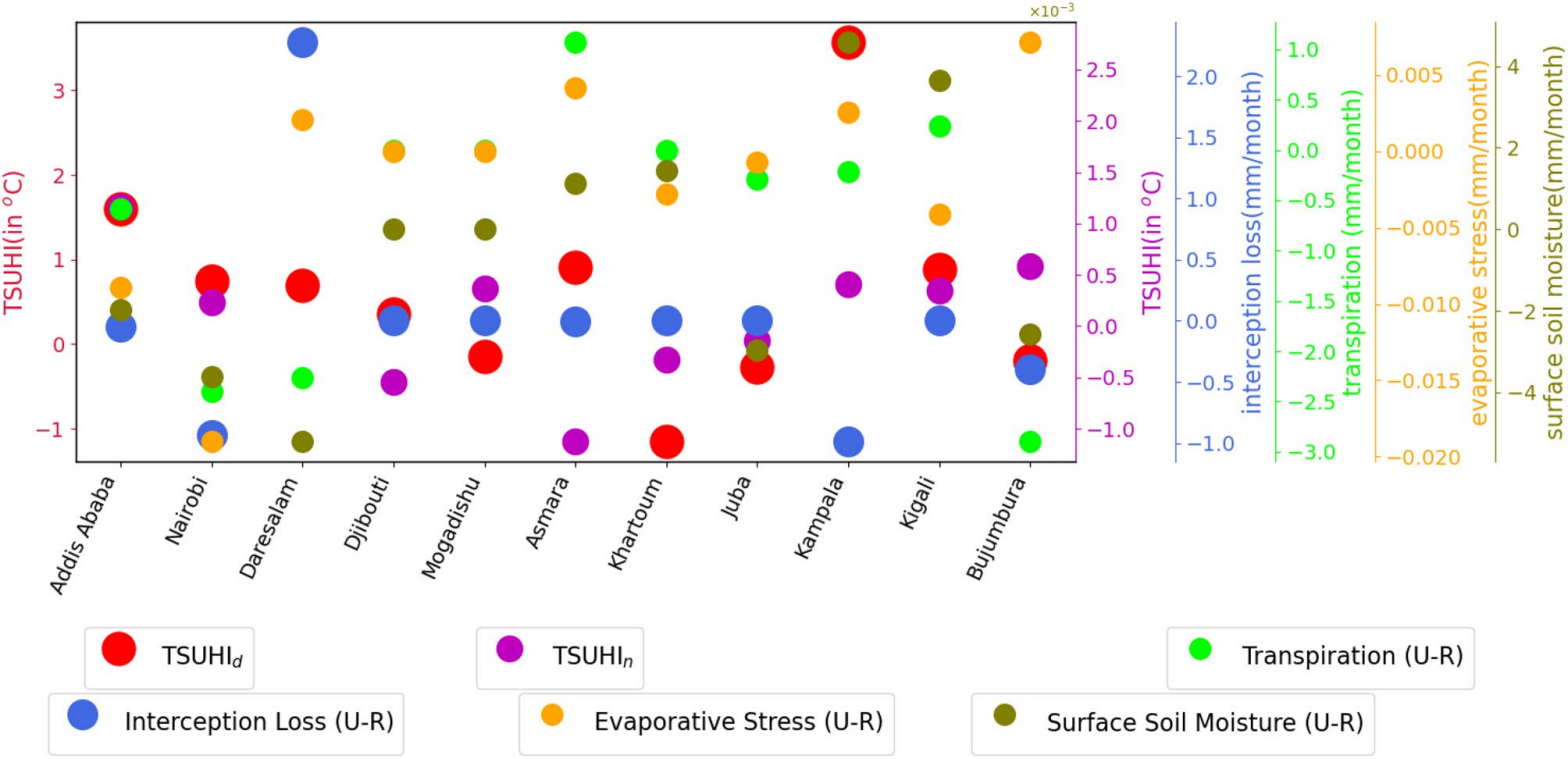
Urban soil moisture and transpiration affecting the tropical surface urban heat islands (TSUHIs) in the east Africa region. Different sizes and colors of circular objects are used to represent the different variables. The large circles with the red and pink colors represent tropical surface urban heat islands during the day and night respectively, the blue, light green, orange and dark green colors represent the interception loss, transpiration, evaporative stress, and surface soil moisture differences between urban and rural areas respectively. The y-axis line colors and labels have similar colors with the circles representing each of the variables. The y-axis for TSUHI$_{n}$, interception loss, transpiration, evaporative stress and soil moisture are shown on the right side. The x-axis shows the corresponding capital cities in the east Africa region.

**Figure 11:**
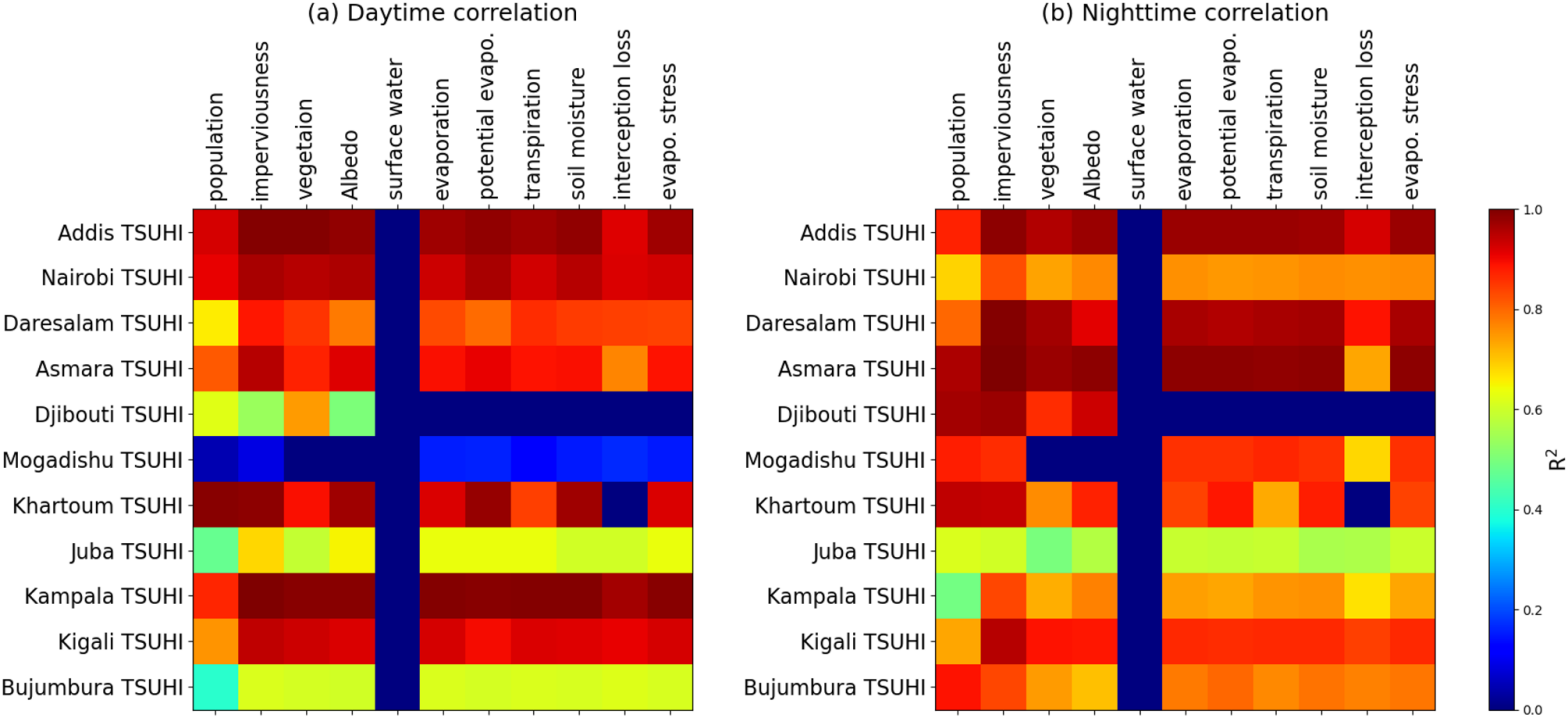
The R$^{2}$ values of the correlation between tropical surface urban heat islands (TSUHIs) in the Greater Horn of Africa (GHA) region with the associated factors during (**a**) day and (**b**) night times.

The background climate affecting the urban heat islands are evaporation, transpiration and soil moisture. Soil moisture is taken as an essential climate variable^50^ because it has a direct impact on the climate system through atmospheric feedback. It is the source of moisture for evapotranspiration over the dry lands, and is involved in both the hydrologic and energy cycles. Evaporation is an essential climate variable as it regulates the thermal environment. Cities with high evaporation rate than the surrounding rural areas, such as Daresalam and Bujumbura (Fig. 9) have a relatively low diurnal tropical surface urban heat islands. There is a strong correlation between evaporation and the TSUHIs of most cities (Fig. 11a and b). Few cities, such as Djibouti, Mogadishu, Juba and Bujumbura during the day (Fig. 11a) and only Juba during the night (Fig. 11b), have shown weak correlation between evaporation and TSUHIs. These cities also do not have sufficient soil moisture required for evaporation. Transpiration is also weak for these cities. The soil moisture and evaporation has similar association with the TSUHIs. Such results are expected because in arid and semi-arid environments, when there isn’t sufficient moisture, the evaporation intensity is lower. This would exacerbate the already dry environments by overheating further. Transpiration is an important factor that reduces urban over heating. In cities where the urban vegetation is relatively higher, e.g., Asmara (Fig. 10), the TSUHI intensity is reduced. The correlation between TSUHIs and transpiration in each of the cities are strong in most of the cities except Juba and Kigali during the day and Juba only during the night (Fig. 11b).

## Conclusion

Results from this study indicated that the capital cities in tropical east Africa exhibited urban surface heat islands that ranges from 1 $^{\circ }$C to 8 $^{\circ }$C depending upon the city size, surface properties and background climate. The tropical surface urban heat islands varies diurnally and seasonally for each city type. In almost all of the cities, the tropical surface urban heat islands during the night is lower that during the day. High intensity tropical surface urban heat islands are manifested from late summer to winter. But, the seasonal characteristics of the tropical urban heat islands varies from one city to the other depending upon its surface features and background climate.

Spatially, some cities have distinct urban heat islands in the center of the cities similar to well developed urban areas in mid- and high latitudes. The second type of cities have tropical surface urban heat islands in the opposite side of the water bodies. This is as a result of the cooling effect of sea-breeze circulation during the day, where the air is cooler near the water bodies than further away from the rivers. The opposite happened during the night, that is part of the cities near the water are warmer than further away during the night. A good example for this urban characteristics is Asmara city. The third category of the cities have inhomogeneous distribution of tropical surface urban heat islands. This inhomogeneity is expected because most of the developed areas in these tropical cities are not uniformly distributed. That is high rise buildings are mixed with low rise buildings and slums.

The regional contribution of the tropical surface urban heat islands is to enforce more warming exacerbating the drought impacts. Out of the 12 cities considered in this study, 10/8 have positive contribution to the regional climate during the day/night respectively. The mean regional contribution from these cities to the regional climate are 0.64 $^{\circ }$C/0.34 $^{\circ }$C during the day/night respectively. The maximum contribution, 4.8 $^{\circ }$C came from Kampala during the day and 3.9 $^{\circ }$C from Daresalam during the night. These two cities are well developed where their urban size and surface properties are the main contributors to the regional warming. This additional warming exacerbates the dry urban and rural environments, worsening the drought effects in these tropical urban areas. This has a direct effect on the availability and quality of water required for irrigation and hygiene.

Different surface factors and background climates played different roles in these east African capital cities. The surface factors associated with the tropical surface urban heat islands are urban population, impervious and vegetation fractions, total albedo, and surface water distribution. The background climate properties are evaporation, transpiration, and soil moisture. In Addis Ababa, Nairobi, Khartoum and Kampala, all factors except surface water affects the tropical surface urban heat islands. In Daresalam, impervious and vegetation fractions, evaporation and transpiration affects the tropical surface urban heat island. In Asmara, all factors except interception loss, in Djibouti, vegetation, in Mogadishu, all factors show low contribution, in Juba, imperviousness, in Kigali, all factors except population, in Bujumbura, weak correlation with all factors (Fig. 11a). Similarly, during the night different associations with different cities are observed (Fig. 11b). This implies that each city has its own unique characteristics that relates its surface urban heat islands with the surface factors and the background climate.

This study therefore recommends that cities in the tropical regions must prepare the city developments to reduce the impacts of the extremes of heat in the regions. It is because the region exhibits similar or worse urban surface heat islands compared to the subtropical, mid-latitude and high-latitudinal regions. Unlike, the mid- to high-latitudes, the causes of the tropical surface urban heat islands is not highly dependent on impervious to vegetation fractions only. Rather, it is dependent on the availability of surface moisture and evapotranspiration. This study didn’t take into account the inter-city variation based on the local climate zones because the urban development in tropical east Africa is highly heterogeneous. It also did not consider the impacts of sea-breezes, mountain breezes and other secondary circulations because these factors require extended studies focusing on these specific circulations and their impacts on urban climate. More studies incorporating the different local climate zones, sea-breeze circulations and other surface factors are needed. The climatological interaction of the tropical surface urban heat islands with droughts also requires further investigation.

## Data Availability

The MODIS land surface temperature and urban land fraction; the SPOT/VGTand PROBA-V surface albedo; the Space Shuttle Topographic Mission (SRTM) and MODIS; and the Global Land Evaporation Amsterdam Model (GLEM) data used in this study are obtained from the Integrated Climate Data Center (ICDC, icdc.cen.uni-hamburg.de) University of Hamburg, Hamburg, Germany. It is available freely for anyone from https://www.cen.uni-hamburg.de/en/icdc.html. The stations temperature are obtained from the Berkley Earth Data https://berkeleyearth.org/data/. The population data is obtained from the World Population Review https://worldpopulationreview.com/.
